# VariScreen secures the screening of high-risk varices in patients with hepatitis B virus-related cirrhosis beyond Baveno VI criteria

**DOI:** 10.3389/fphys.2022.1006657

**Published:** 2022-09-27

**Authors:** Min Tan, Wuxiang Zhang, Hong Zhou, Yujuan Liu, Tao Lu, Yin Zhang, Chuan Li, Yanyan Yang, Yunchong Wu, Han Hu, Ying Li, Fangwan Yang, Shide Lin

**Affiliations:** ^1^ Department of Infectious Diseases, Affiliated Hospital of Zunyi Medical University, Zunyi, China; ^2^ Department of Infectious Diseases, Suining Central Hospital, Suining, China; ^3^ Department of Infectious Diseases, The Third Affiliated Hospital of Zunyi Medical University (The First People’s Hospital of Zunyi City), Zunyi, China; ^4^ Department of Infectious Diseases, The Second Affiliated Hospital of Zunyi Medical University, Zunyi, China; ^5^ College of Laboratory Medicine, Zunyi Medical University, Zunyi, China

**Keywords:** Baveno VI criteria, high-risk varices, hepatitis B, cirrhosis, platelets, liver stiffness measurement

## Abstract

We aimed to validate the performance of the ratio of the platelet count (PLT) to liver stiffness measurement (LSM) in excluding high-risk varices (HRVs) in patients with hepatitis B virus (HBV)-related compensated cirrhosis beyond Baveno VI criteria. A total of 310 patients were assessed. The performances of the PLT:LSM ratio (PLER), PLER adjusted for the international normalized ratio, etiology, age, and sex (PLEASE), and the sequential algorithm for HRV screening (VariScreen) in excluding HRVs were evaluated and compared with those of expanded Baveno VI criteria (LSM <25 kPa and PLT >110×10^9^/L, EB6C); PLT >150×10^9^/L and model for end-stage liver disease score = 6 (P150M6 criterion); PLT >120×10^9^/L and albumin >36 g/L (P120A36 criterion); and albumin-bilirubin (ALBI) grade and PLT score (ALBI-PLT score). Among the enrolled patients, 43 (13.9%) had HRVs. The area under the receiver operating characteristic curve of PLER for predicting HRVs (0.771, 95% confidence interval, 0.720–0.817) was significantly higher than that for PLT and LSM (*p* < 0.01). PLER was an independent risk factor for HRVs. VariScreen, PLEASE, and PLER could spare 20 (6.5%), 91 (29.4%), and 60 (19.4%) endoscopies, with 0, 3 (3.3%), and 1 (1.7%) HRVs missed, respectively. The EB6C and P120A36 criteria could spare 45 (14.5%) and 36 (11.6%) endoscopies, with 1 (2.2%) and 1 (2.8%) HRVs missed, respectively. The P150M6 criterion and ALBI-PLT score missed 6.8% and 10.3% of HRVs, respectively. We found that PLER performed better than other non-invasive tests. VariScreen secured the screening of HRVs in patients with HBV-related cirrhosis beyond Baveno VI criteria.

## Introduction

Patients with decompensated liver cirrhosis have a much worse prognosis than patients with compensated liver cirrhosis. Esophagogastric varices (EV) and esophagogastric variceal bleeding (EVB) are common manifestations of hepatic decompensation (Garcia-Tsao et al., 2007). Early identification of patients with high-risk varices (HRVs) and prevention of EVB are important for preventing hepatic decompensation and improving the survival chances of patients with liver cirrhosis (Tsochatzis et al., 2014).

Endoscopy is the reference method for identifying HRVs (Yoshiji et al., 2021). Recently, the Baveno VII consensus recommended the use of beta-blockers in patients with compensated advanced chronic liver diseases (cACLDs) and clinically significant portal hypertension (CSPH), and endoscopy may not be required in patients taking beta-blockers (de Franchis et al., 2022). However, the “gold standard” for diagnosing CSPH is the measurement of the hepatic venous pressure gradient (HVPG). The HVPG requires highly trained technicians and cannot be carried out in most hospitals in China and other developing countries. Therefore, endoscopy is still required for most patients with liver cirrhosis to screen HRVs.

Endoscopy is an invasive examination which is expensive and carries risks to patients (European Association for the study of the Liver, 2015). The Baveno VI consensus recommended that endoscopy can be avoided safely in patients with cACLD or compensated liver cirrhosis with a liver stiffness measurement (LSM) <20 kPa and platelet count (PLT) >150 ×10^9^/L (de Franchis, 2015). These criteria have been validated extensively and found to have high reliability and safety for excluding patients without HRVs; a very low (<5%) risk of missing HRVs has been documented (Zhou et al., 2020). In 2017, the American Association for the Study of Liver Disease recommended using Baveno VI criteria to stratify the EV risk in patients with liver cirrhosis (Garcia-Tsao et al., 2017). However, the major limitation to Baveno VI criteria is that only 8.1–46.2% of endoscopies could be spared; >40% of unnecessary endoscopies cannot be spared in patients with cACLD (Maurice et al., 2016; Augustin et al., 2017a; Bae et al., 2018; Stafylidou et al., 2019). Therefore, improving the performance of non-invasive tests (NITs) in excluding HRVs has garnered considerable interest in recent years.

Many studies have been conducted to improve the performance of the NITs in excluding HRVs (Abraldes et al., 2016), such as expanded Baveno VI criteria (LSM <25 kPa and PLT >110×10^9^/L, EB6C criterion) (Augustin et al., 2017b); PLT >150×10^9^/L and model for end-stage liver disease (MELD) score = 6 (P150M6 criterion) (Jangouk et al., 2017); PLT >120×10^9^/L and albumin (ALB) >36 g/L (P120A36 criterion) (Calvaruso et al., 2019); and ALB-bilirubin (ALBI) grade and PLT score (ALBI-PLT score) (Abd Elbaser et al., 2022). However, great heterogeneity in the performance and safety of these criteria was found in subsequent studies (Bai and Abraldes, 2022). A different HRV prevalence resulting from a different distribution of patients within and beyond Baveno VI criteria was found to be one of the major causes of this heterogeneity (Bai and Abraldes, 2022).

Recently, a screening strategy based on the PLT/LSM ratio (PLER) has been found to be effective for excluding HRVs safely (Berger et al., 2021), but the performance of this screening strategy has not been validated externally. Baveno VI criteria have been accepted for stratifying the EV risk in clinical practice, so improving the performance and safety of these NITs in patients beyond Baveno VI criteria is crucial. In this study, we aimed to validate the performance of PLER and compare it with that of other NITs for excluding HRVs in patients with hepatitis B virus (HBV)-related compensated cirrhosis beyond Baveno VI criteria.

## Methods

### Inclusion criteria

The inclusion criteria were as follows: 1) HBV infection, 2) compensated cirrhosis, 3) transient elastography (TE) and endoscopy undertaken within 3 months, and 4) LSM ≥20 kPa and/or PLT ≤150×10^9^/L.

### Exclusion criteria

The exclusion criterion was at least one of the following: 1) other causes of cirrhosis except HBV infection (infection with the hepatitis C virus, alcoholic liver disease, and primary biliary cirrhosis), liver cancer, or other malignancies; 2) interventional treatment for the complications of portal hypertension; 3) previous splenectomy; 4) prior treatment with non-selective beta-blockers; 5) pregnancy; 6) other serious injuries to organs; and 6) infection with the human immunodeficiency virus.

### Study population

**FIGURE 1:**
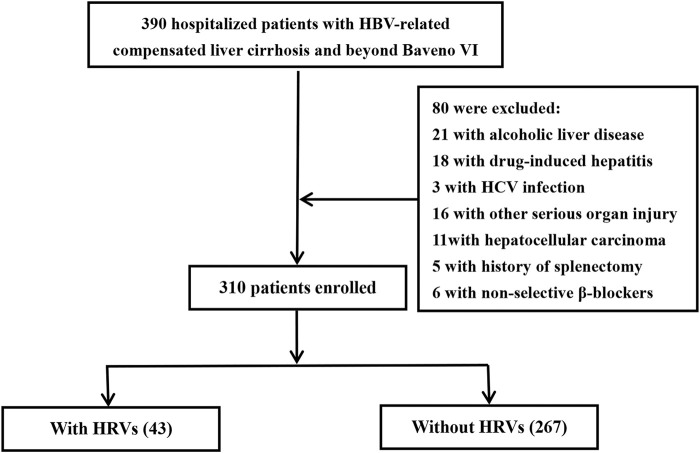
Protocol for screening and case selection. Patients beyond Baveno VI criteria had a liver stiffness measurement ≥20 kPa and/or platelet count ≤150 × 10^9^/L. HCV, hepatitis C virus; HRVs, high-risk varices.

Patients with HBV-related compensated cirrhosis admitted to Suining Central Hospital (Suining, China) or the Affiliated Hospital of Zunyi Medical University (Zunyi, China) from September 2014 to April 2022 were reviewed retrospectively. A total of 390 patients were recruited, and finally, 310 patients were included in the analysis (Figure 1).

### Clinical variables

Patient characteristics at baseline were collected, including demographic data and laboratory parameters (international normalized ratio (INR), PLT, white blood cell count and levels of ALB, alanine transaminase (ALT), aspartate transaminase (AST), blood urea nitrogen, creatinine (Cr), gamma-glutamyl transpeptidase (GGT), and globulin). If patients had multiple measurements of laboratory parameters, we used the results closest in time to the TE procedure.

Cirrhosis was diagnosed by liver biopsy or as a composite of clinical signs and the findings of endoscopy, imaging, and laboratory tests. “Decompensated cirrhosis” was defined as the presence of EVB, ascites, or new hepatic encephalopathy.

The MELD score was calculated according to the following formula (Asrani and Kamath, 2015):

MELD=3.8×ln[total bilirubin (TBil in mg/dL)]+11.2×ln(INR)+9.6×ln[Cr (mg/dL) ]+6.4×(constant for liver disease etiology: 0 if cholestatic or alcoholic, otherwise 1).

Gastroscopic data were collected. The EVB risk was judged by using the varix stage (none, small, medium, and large). “HRVs” were defined as medium/large varices or small varices with red wale marks.

The LSM by TE used a FibroScan™ device (Echosens, Paris, France). The LSM was taken in the fasting state and expressed in kPa. As active hepatitis in patients with liver cirrhosis could elevate LSM (Shiha et al., 2017), we performed TE in patients with active hepatitis after their acute liver inflammation resolved. Measurements were made by experienced operators using an M probe. The LSM was considered to be successful if 10 valid measurements were obtained with a success rate >60% and if the interquartile range-to-median ratio (IQR/M) <0.3.

### Non-invasive tests of high-risk varices

PLER was calculated as PLT (×10^9^)/LSM (kPa) (Berger et al., 2021). “PLEASE” is PLER adjusted based on etiology, age, sex, and the INR. “VariScreen” is a sequential algorithm for varices screening by PLT, LSM, and the INR. PLER, PLEASE, and VariScreen were calculated from a calculator available at: http://forge.info.univ-angers.fr/wgh/wstat/pler-please-variscreen. php/.

The EB6C criteria were LSM <25 kPa and PLT >110×10^9^/L (Augustin et al., 2017b). The P150M6 criteria were PLT >150×10^9^/L and PLT <150×10^9^/L plus MELD score = 6 (Jangouk et al., 2017). The P120A36 criteria were PLT >120×10^9^/L and ALB >36 g/L (Calvaruso et al., 2019).

The ALBI score was calculated using the following formula (Abd Elbaser et al., 2022): ALBI score = −0.085 × (ALB, g/L) + 0.66 × log(TBil, μmol/L). The ALBI score was graded as follows: ALBI-1 if ≤ −2.60, ALBI-2 if −2.59 to −1.39, and ALBI-3 if > −1.39 (Bai and Abraldes, 2022). The ALBI-PLT score was calculated by adding the ALBI grade and PLT. The cutoff value for PLT was 150×10^9^/L. One point was given if PLT was >150×10^9^/L, and two points were given if ≤ 150×10^9^/L. The ALBI-PLT score was the sum of the ALBI grade and the point of PLT. An ALBL-PLT score ≤3 was graded as a “low risk” of HRV.

### Statistical analyses

Data analyses were undertaken using SPSS 19.0 (IBM, Armonk, NY, United States). For patients with HRVs and patients without HRVs, comparison of characteristics at baseline was conducted using χ^2^ tests for categorical variables, *t*-tests for variables with a normal distribution, and Mann–Whitney *U* tests for variables with an abnormal distribution. The logistic regression analysis was used for univariate and multivariate analyses. MedCalc 15.8 (www.medcalc.org) was used to calculate receiver operating characteristic (ROC) curves. The accuracy of each diagnostic criterion was evaluated according to the area under the ROC curve (AUROC). AUROC values were compared using the DeLong test. To assess the accuracy of NITs, we used the sensitivity, specificity, positive prediction value (PPV), and negative predictive value (NPV) for ruling out HRVs. Missed HRVs/spared endoscopies were calculated to reflect the safety of NITs for excluding HRVs.

## Results

### Clinical characteristics of patients with hepatitis B virus-related cirrhosis beyond Baveno VI criteria

Among the 310 patients enrolled in our study, 105 (33.9%) patients had EVs, 62 (20.0%) had small EVs without red wale signs, and 43 (13.9%) had HRVs (medium or large EV). The MELD score was 10.3 ± 3.8, with 125 (40.3%) patients having a MELD score ≥10.0, 199 patients (64.2%) being graded as class A, and 108 (34.8%) patients being graded as class B in the Child–Pugh–Turcotte system.

Of the 310 patients, 185 (37 with HRVs) did not meet Baveno VI criteria due to having both LSM ≥20 kPa and PLT ≤150×10^9^/L, 30 (two with HRVs) did not meet Baveno VI criteria due to having only LSM ≥20 kPa, and 95 (four with HRVs) did not meet Baveno VI criteria due to having only PLT ≤150×10^9^/L. HRV prevalence in patients who did not meet both LSM and PLT criteria was significantly higher than that in patients who did not meet either the LSM criterion or PLT criterion (*p* < 0.01).

PLER, the INR, and the LSM score were significantly higher in patients with HRVs, whereas levels of ALT, AST, GGT, and PLT were significantly lower in those who did not have HRVs (Table 1).

**TABLE 1:** Baseline characteristics of patients with HBV-related cirrhosis with/without HRVs beyond Baveno VI criteria.

Variable	Without HRVs (*n* = 267)	With HRVs (*n* = 43)	t/u/χ^2^	*p*
Age (years)	47.5 ± 11.4	47.3 ± 11.5	0.099	NS
Male	218 (81.6%)	40 (93.0%)	3.433	NS
Bodyweight (kg)	63.9 ± 9.5	67.6 ± 7.4	−1.541	NS
BMI (kg/m^2^)	23.0 ± 3.6	23.6 ± 1.9	−2.063	NS
NUC	63 (23.6%)	13 (30.2%)	0.882	NS
ALT (U/L)	100.3 (54.0–270.0)	51.7 (33.0–81.0)	−4.294	0.000
AST (U/L)	86.7 (52.0–191.0)	54.0 (37.0–87.0)	−3.426	0.001
GGT (U/L)	111.0 (61.0–196.0)	70.2 (37.0–119.0)	−3.060	0.002
TBil (μmol/L)	26.2 (16.9–45.2)	24.8 (15.5–44.2)	−0.489	NS
ALB (g/L)	35.8 ± 5.5	34.9 ± 5.8	0.914	NS
GLB (g/L)	32.6 ± 21.7	33.0 ± 7.0	−0.132	NS
AFP (ng/ml)	19.8 (6.7–91.1)	14.6 (5.9–47.8)	−0.630	NS
BUN (mmol/L)	4.4 (3.6–5.2)	4.5 (3.8–5.4)	−0.798	NS
Cr (μmol/L)	73.0 (65.0–80.0)	74.0 (68.0–85.0)	−1.202	NS
INR	1.0 (0.9–1.2)	1.2 (1.0–1.4)	−3.461	0.001
WBC (10^9^/L)	4.4 ± 1.5	4.0 ± 2.1	1.199	NS
PLT (10^9^/L)	95.0 (66.0–127.0)	65.0 (53.0–88.0)	−4.153	0.000
LSM (kPa)	24.2 (16.9–33.8)	34.3 (23.0–45.7)	−3.887	0.000
PLER	4.97 ± 3.91	2.46 ± 1.69	4.141	0.000
CPT score	6.2 ± 1.2	6.2 ± 1.2	0.153	NS
MELD score	10.2 ± 3.9	10.7 ± 3.5	−0.813	NS
MELD score ≥10	109 (40.8)	24 (55.8)	3.386	NS

Data include *n*, %, mean ± SD, or median (interquartile range).

AFP, alpha fetoprotein; ALB, albumin; ALT, alanine aminotransferase; AST, aminotransferase; BMI, body mass index; GGT, gamma-glutamyl transpeptidase; BUN, blood urea nitrogen; Cr, creatinine; CTP, Child–Pugh–Turcotte; GLB, globulin; HRVs, high-risk varices; INR, international normalized ratio; LSM, liver stiffness measurement; PLER, platelet count: LSM ratio; PLT, platelet count; MELD, model of end-stage liver disease; NUCs, nucleoside/nucleotide analogs; TBil, total bilirubin; WBC, white blood cell.

### Comparison of the platelet count, liver stiffness measurement, and PLER for identifying high-risk varices in patients with hepatitis B virus-related cirrhosis beyond Baveno VI criteria

**FIGURE 2:**
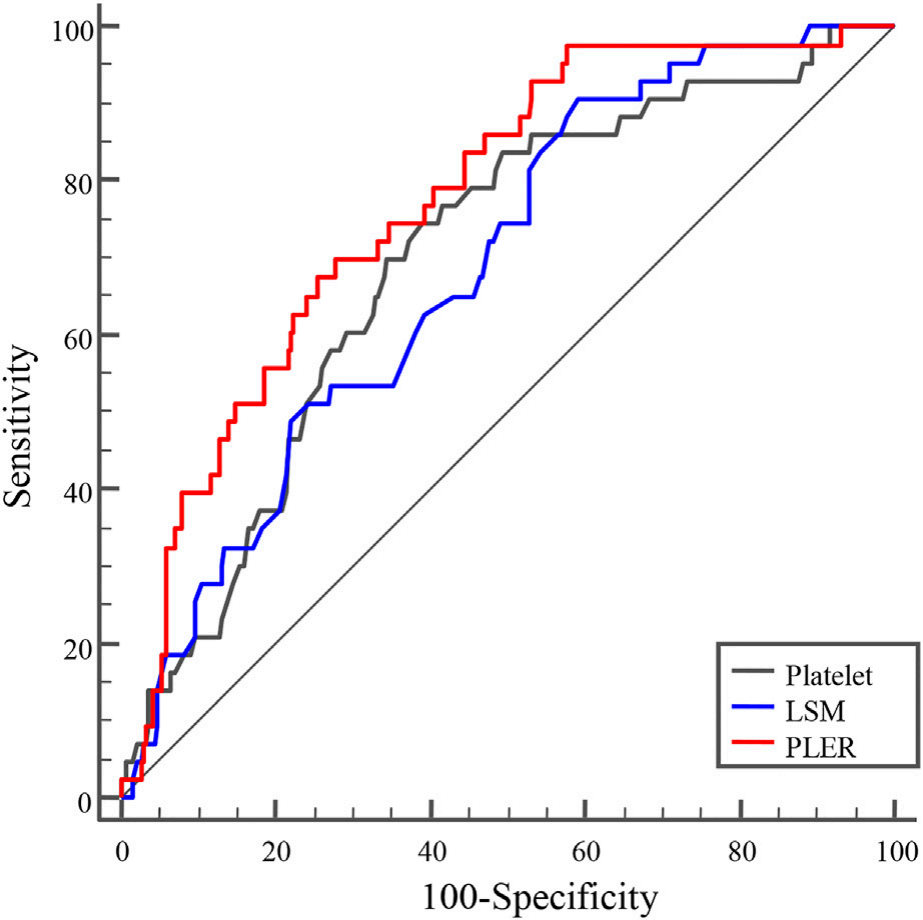
AUROC curves of PLER, PLT, and LSM for diagnosing HRVs in patients with HBV-related cirrhosis beyond the Baveno VI criteria. AUROCs of PLER, PLT, and LSM were 0.771 (95% confidence interval [CI]: 0.720–0.817), 0.697 (95%CI: 0.643–0.748), and 0.685 (95%CI: 0.630–0.736), respectively. AUROC, area under receiver operating characteristic curve; HRVs, high-risk varices; LSM, liver stiffness measurement; PLER, platelet count: liver stiffness measurement ratio; PLT, platelet count.

Next, we evaluated the diagnostic value of PLT, LSM, and PLER for identifying HRVs in patients beyond Baveno VI criteria. PLER had a significantly higher AUROC (0.771, 95% confidence interval [CI]: 0.720–0.817) than PLT (0.697, 95% CI: 0.643–0.748) and LSM (0.685, 95% CI: 0.630–0.736) (*p* < 0.01) (Table 2 and Figure 2). At a cutoff of 2.72, PLER had a sensitivity of 69.8%, specificity of 72.3%, PPV of 28.8%, NPV of 93.7%, positive likelihood ratio of 2.52, and negative likelihood ratio of 0.42.

**TABLE 2:** Performance of PLT, LSM, and PLER for diagnosing HRVs in patients with HBV-related liver cirrhosis beyond Baveno VI criteria.

	AUROC (95%CI)	Cutoff	Se (%)	Sp (%)	PPV (%)	NPV (%)	+LR	−LR
PLT	0.697 (0.643–0.748)	81	72.1	62.6	23.7	93.3	1.92	0.45
LSM	0.685 (0.630–0.736)	21.1	90.7	40.8	19.8	96.5	1.53	0.23
PLER	0.771 (0.720–0.817)	2.72	69.8	72.3	28.8	93.7	2.52	0.42

AUROC, area under the receiver operating characteristic curve; CI, confidence interval; HRVs, high-risk varices; LSM, liver stiffness measurement; NPV, negative predictive value; PLER, platelet count: LSM ratio; PLT, platelet count; Se, sensitivity; Sp, specificity; PPV, positive predictive value; +LR, positive likelihood ratio; −LR, negative likelihood ratio.

### Risk factors associated with high-risk varices in patients with hepatitis B virus-related cirrhosis beyond Baveno VI criteria

The univariate analysis showed that risk factors for HRVs in patients beyond Baveno VI criteria were the INR, PLT, ALT level, LSM, and PLER score (Table 3). PLER was the PLT:LSM ratio, so we included the INR, ALT level, and PLER in the multivariate analysis. We found that PLER (odds ratio (OR) = 0.634, 95% CI: 0.501–0.803) and ALT level (OR = 0.993, 95% CI: 0.988–0.997) were independent risk factors associated with HRVs in patients with HBV-related compensated cirrhosis beyond Baveno VI criteria.

**TABLE 3:** Univariate and multivariate analyses of risk factors associated with HRVs in patients with HBV-related liver cirrhosis beyond Baveno VI criteria.

Variable	Univariate analysis				Multivariate analysis			
	*β*	OR	95% CI	*p*	*β*	OR	95% CI	*p*
INR	2.349	10.474	2.297–47.479	0.002				
ALT	−0.007	0.993	0.988–0.997	0.001	−0.007	0.993	0.988–0.997	0.002
LSM	0.038	1.039	1.017–1.061	0.000				
PLT	−0.020	0.980	0.971–0.990	0.000				
PLER	−0.520	0.594	0.474–0.746	0.000	−0.456	0.634	0.501–0.803	0.000

ALT, alanine aminotransferase; CI, confidence interval; HBV, hepatitis B virus; HRVs, high-risk varices; INR, international normalized ratio; LSM, liver stiffness measurement; PLER, platelet count: LSM ratio; PLT, platelet count.

### Use of PLER for excluding high-risk varices in patients with hepatitis B virus-related cirrhosis beyond Baveno VI criteria

Next, we explored the performance of PLER, PLEASE, and VariScreen according to the cutoff values recommended by Arthur and colleagues (Berger et al., 2021). We compared their performances with those of the EB6C criterion, P120A36 criterion, P150M6 criterion, and ALBI-PLT score in excluding HRVs in patients with HBV-related compensated cirrhosis beyond Baveno VI criteria.

VariScreen had the highest sensitivity (100%) and NPV (100%) among all NITs (Table 4). VariScreen could spare 20 (6.5%) patients from having endoscopies without HRVs being missed. PLEASE could spare 91 (29.4%) patients from having endoscopies, with missing HRVs in three (3.3%) patients. Among three patients missed by PLEASE, two patients had large varices with a PLT of 93×10^9^/L and 125×10^9^/L; and an LSM of 21.5 and 12.0 kPa, respectively, and one patient had a medium varix with a PLT of 161×10^9^/L and LSM of 34.8 kPa. PLER could spare 60 (19.4%) patients from having endoscopies, with missing HRVs in one (1.7%) patient. EB6C could spare 45 (14.5%) patients from having endoscopies, with missing HRVs in one (2.2%) patient. PLER and the EB6C criterion missed the same patient with PLT = 125×10^9^/L, LSM of 12 kPa, and a large varix. The P120A36 criterion could spare 36 (11.6%) patients from having endoscopies, with missing HRVs in one (2.8%) patient with PLT = 151×10^9^/L, ALB = 42 g/L, and a large varix.

**TABLE 4:** Performances of the PLT:LSM ratio and NITs for excluding HRVs in patients with HBV-related compensated cirrhosis beyond Baveno VI criteria.

Criterion	Se	Sp	PPV	NPV	Spared endoscopy	Missed HRVs/spared endoscopy
EB6C	97.7	16.5	15.8	97.8	45/310 (14.5%)	1/45 (2.2%)
P150M6	88.4	25.8	16.1	93.2	74/310 (23.9%)	5/74 (6.8%)
P120A36	97.7	13.1	15.3	97.2	36/310 (11.6%)	1/36 (2.8%)
ALBI-PLT score	81.4	26.2	15.1	89.7	78/310 (25.2%)	8/78 (10.3%)
PLEASE	93.0	32.9	18.3	96.7	91/310 (29.4%)	3/91 (3.3%)
VariScreen	100	7.49	14.8	100	20/310 (6.5%)	0/20 (0%)
PLER	97.7	22.10	16.8	98.3	60/310 (19.4%)	1/60 (1.7%)

ALBI-PLT: albumin–bilirubin (ALBI) grade and the platelet count (PLT) score; EB6C, expanded Baveno VI criterion; HRVs, high-risk varices; NITs, non-invasive tests; NPV, negative predictive value; P120A36 criterion, PLT >120 × 10^9^/L and albumin >36 g/L; P150M6 criterion, PLT >150 × 10^9^/L and model for end-stage liver disease (MELD) score = 6; PLER, PLT:LSM ratio; PLEASE, PLER adjusted for etiology, age, sex, and international normalized ratio (INR); VariScreen, a sequential algorithm for varices screening by PLT, liver stiffness measurement, and the INR; PPV, positive predictive value; Se, sensitivity; Sp, specificity.

The P150M6 criterion and ALBI-PLT score had low sensitivity and NPV. Although the use of the P150M6 criterion could spare 74 to 78 patients from having endoscopies, HRVs would be missed in 6.8% and 10.3% of cases, respectively.

## Discussion

Recently, the Baveno VII consensus has recommended the use of beta-blockers for patients with cACLD and CSPH. It also recommended using LSM ≥25 kPa to help diagnose CSPH and LSM ≤15 kPa plus PLT ≥150×10^9^/L to exclude CSPH (de Franchis et al., 2022). However, in clinical practice, most patients with compensated liver cirrhosis cannot be categorized by these criteria. Moreover, in patients with contraindications or intolerance to beta-blockers, endoscopic assessment of HRVs is required unless NITs suggest a very low risk of HRVs. Baveno VI criteria have been validated and accepted for HRV stratification, so excluding HRVs by NITs in patients beyond Baveno VI criteria is important for improving the management of liver cirrhosis.

In this study, we found that PLER, the INR, and the LSM score were significantly higher in patients with HRVs, whereas levels of ALT, AST, GGT, and PLT were significantly lower than in those who did not have HRVs. It is difficult to explain the results of lower ALT, AST, and GGT levels in patients with HRVs than those in patients without HRVs. One of the possible explanations was that we included hospitalized patients beyond Baveno VI criteria in this study; although we performed TE after acute liver inflammation was resolved, most of the patients still had mild liver inflammation at the time of performing TE. As liver inflammation could elevate LSM and make the patients with high ALT difficult to fulfill the Baveno VI criteria (Shiha et al., 2017), patients with high ALT had a lower prevalence of HRVs than patients with low ALT. As a result, patients without HRVs had higher levels of ALT, AST, and GGT than patients with HRVs. Indeed, in this study, we also discovered that ALT was a negative predictor of HRVs.

Patients with compensated liver cirrhosis include those who are within and beyond Baveno VI criteria. Most studies have included patients who are within and beyond Baveno VI criteria to verify the efficacy and safety of NITs in excluding HRVs. Because patients who are within Baveno VI criteria had a significantly lower prevalence of HRVs than patients who were beyond Baveno VI criteria, the different distribution of patients within and beyond Baveno VI criteria resulted in a different HRV prevalence (Zhou et al., 2019; Hu and Wen, 2021; Calès et al., 2022). A recent meta-analysis found that a different HRV prevalence resulted in different performances of NITs (Bai and Abraldes, 2022). In addition, the performances of NITs are affected by the causes of liver cirrhosis (Berger et al., 2021). In this study, we included only patients with HBV-related cirrhosis beyond Baveno VI criteria to compare the performances of PLER with those of other NITs. These characteristics of patients overcame the bias elicited by including patients with different causes of liver cirrhosis and the different distribution of patients within and beyond Baveno VI criteria. Therefore, our results are more comparable than those in the previous studies.

We found that PLER had a significantly higher AUROC than that of PLT or LSM. PLER and PLEASE could spare more patients from having endoscopies than the use of EB6C and P120A36 criteria. These results demonstrated that PLER had a higher ability than PLT or LSM for identifying HRVs. PLER and PLEASE performed better than EB6C and P120A36 criteria for stratifying the EV risk in patients with HBV-related cirrhosis beyond Baveno VI criteria. The P150M6 criterion and ALBI-PLT score had missed HRVs >5%, which suggested that they were not safe for excluding HRVs in patients beyond Baveno VI criteria.

Another major finding in our study was that VariScreen secured screening without HRVs being missed in patients with HBV-related compensated liver cirrhosis beyond Baveno VI criteria. VariScreen was established by Berger et al. (2021). They retrospectively studied data from 2,368 patients with chronic liver disease of different causes. They found the ratio of PLT to LSM could predict HRVs because it provided a single cutoff value for a fixed prevalence of missed HRVs. PLER interacted significantly with etiology, sex, and the INR, so these variables were adjusted to produce PLEASE. The latter was used to screen HRVs. VariScreen comprised three steps. First, the HRV risk was excluded in patients with PLT >402 × 10^9^/L and LSM < 9 kPa. Second, if PLER ≥17, then an endoscopy was unnecessary. Third, if PLER <6.2, then an endoscopy was indicated. A PLER score ≥6.2 and PLER score < 17 necessitated a PLEASE calculation.

VariScreen performed well in patients with chronic liver disease of any cause or severity (Berger et al., 2021). It spared 34.5% of patients from endoscopies with HRVs missed in 2.9% of patients (Baveno VI criteria spared 23.9% of patients with missing HRVs in 2.9%). Moreover, VariScreen performed well without HRVs being missed in patients with a MELD score >10.0. However, the performance of VariScreen in patients with HBV-related liver cirrhosis beyond Baveno VI criteria was not validated. In our study, 125 (40.3%) patients had a MELD score ≥10.0. VariScreen had the highest sensitivity and NPV among all NITs evaluated in our study. Although VariScreen spared only 20 patients from having endoscopies, no HRVs were missed. These results demonstrated that VariScreen had high security for HRV screening in patients with HBV-related cirrhosis beyond Baveno VI criteria.

Our study had two main strengths. First, we made an external validation on the performance of PLER in patients with HBV-related cirrhosis who were beyond Baveno VI criteria. Second, we included only patients beyond Baveno VI criteria to compare the performance of NITs in excluding HRVs. Our results were more comparable than those in studies that included patients within and beyond Baveno VI criteria and with considerable diversity in the cause of liver cirrhosis.

Our study had three main limitations. First, it was a two-center, retrospective study based on LSM assessed and gastroscopies undertaken by different operators (although all of these operators were experienced). Second, the acceptable threshold of the prevalence of missed HRVs in patients within and beyond Baveno VI criteria was defined as <5%. Patients who were within Baveno VI criteria had been excluded from endoscopies after Baveno VI criteria had been accepted for clinical use, so we could not define the acceptable threshold of missed HRVs in patients beyond Baveno VI criteria. However, our results showed that even taking the strictly defined criterion of no missed HRVs, VariScreen could secure screening of HRVs in patients beyond Baveno VI criteria. Third, we included only Asian patients with HBV-related cirrhosis. The generalizability of our results to that of other ethnicities and etiologies remains to be validated.

## Conclusion

We compared, for the first time, the performance of PLER with other NITs in patients with HBV-related cirrhosis beyond Baveno VI criteria. PLER (i.e., the ratio of PLT to LSM) performed better than other NITs. VariScreen secured HRV screening in patients with HBV-related cirrhosis beyond Baveno VI criteria.

## Data Availability

The raw data supporting the conclusions of this article will be made available by the authors, without undue reservation.
